# *Lactobacillus rhamnosus* GG mitigates bone loss induced by mechanical unloading via regulation of the gut-bone axis

**DOI:** 10.3389/fnut.2025.1734220

**Published:** 2026-02-02

**Authors:** Xuezhi Qin, Yu-E Lian, Hanqin Tang, Xin Chai, Yuhai Gao, Yanchun Ma, Jing Guo, Hongli Wang, Yan Wang, Biaomeng Wang, Jiayu Chen, Yixuan Wang

**Affiliations:** 1 Department of Gastroenterology, The 940th Hospital of Joint Logistics Support Force of Chinese PLA, Lanzhou, China; 2 Graduate School of Gansu University of Traditional Chinese Medicine, Lanzhou, China; 3 Department of Basic Medical Laboratory, The 940th Hospital of Joint Logistics Support Force of Chinese PLA, Lanzhou, China; 4 Department of Emergency, An Ning Attached Medical Area, The 940th Hospital of Joint Logistics Support Force of Chinese PLA, Lanzhou, China

**Keywords:** bone loss, gut-bone axis, *Lactobacillus rhamnosus* GG, mechanical unloading, short-chain fatty acids

## Abstract

**Background:**

Bone loss is a serious complication of mechanical unloading, such as that experienced during spaceflight or prolonged bed rest, and represents a significant clinical concern. Although the gut-bone axis has been implicated in bone homeostasis, its role under unloading conditions remains underexplored.

**Methods:**

In this study, we employed a hindlimb unloading (HU) mouse model to investigate the underlying mechanisms of HU-induced bone loss and the potential protective role of *Lactobacillus rhamnosus* GG (LGG). Gut microbiota (16S rRNA sequencing), short-chain fatty acids (LC–MS/MS), intestinal barrier proteins (ZO-1/Occludin), inflammatory cytokines in bone tissue (TNF-α/IL-1β/IL-10), regulatory T (Treg), bone markers (BALP/OPG/OCN/PINP/CTX), and microarchitecture (Micro-CT) were analyzed.

**Results:**

Hindlimb unloading (HU) disrupted gut microbiota composition, reduced short-chain fatty acids (SCFA)-producing bacteria, and decreased SCFA levels, which was accompanied by reduced expression of ZO-1 and Occludin, elevated circulating LPS levels, and enhanced inflammatory markers in the bone microenvironment. Additionally, the proportion of Treg cells was reduced, which was associated with markers indicative of disrupted bone remodeling. LGG treatment was associated with partial restoration of microbial composition and SCFA levels, accompanied by improved intestinal barrier markers, reduced LPS and inflammatory cytokines, increased Treg proportions, and amelioration of bone microarchitecture.

**Conclusion:**

These findings suggested that LGG may have conferred protection against unloading-induced bone loss, potentially through modulation of the gut microbiota, alterations in SCFA profiles, improvement of intestinal barrier function, and immune regulatory changes involving Treg cells. This work highlighted the therapeutic potential of targeting the gut-bone axis to mitigate bone loss in microgravity or immobilization settings.

## Introduction

Prolonged exposure to microgravity environments, such as during spaceflight, or extended periods of bed rest due to illness, has been strongly associated with bone loss resulting from sustained mechanical unloading (1–3). With the growing demand for space exploration, long-duration space habitation is becoming inevitable. However, during extended space missions, even healthy astronauts lose approximately 1–2% of bone mineral density (BMD) per month at weight-bearing sites such as the hip and lumbar vertebrae. In addition, parameters such as BALP, OCN, PICP, and CTX also undergo significant changes (4–6). Alarmingly, this bone loss can persist for months even after returning to Earth (7). Similarly, patients who are bedridden due to chronic illness often develop disuse osteoporosis as a result of reduced mobility (8). While the association between mechanical unloading and bone loss has been well established, effective therapeutic strategies to prevent or mitigate this condition remain limited and represent a critical challenge.

Recently, the notion of the gut-bone axis has gradually emerged, proposing that the gut microbiota can influence host bone metabolism through multiple mechanisms (9). Short-chain fatty acids (SCFAs), generated by probiotic bacteria, serve as critical mediators of this axis (10). SCFAs not only facilitate the proliferation of beneficial bacteria and preserve gut barrier integrity but also act on the immune system by promoting the expansion of Treg cells (11, 12). Tregs are known to release IL-10 and TGF-β, and suppress osteoclastogenesis, thereby maintaining bone mass (13). In addition, gut microbes can directly modulate intestinal barrier function (14, 15). Dysbiosis or increased gut permeability allows endotoxins such as LPS to enter circulation, triggering chronic inflammation, promoting osteoclast hyperactivity, and accelerating bone loss (16–18). Recent studies have also confirmed that unloading models such as tail suspension induce pronounced inflammatory responses in the bone marrow microenvironment, characterized by elevated expression of pro-inflammatory mediators, disrupting bone remodeling and leading to bone loss (19–21). However, the contribution of the gut-bone axis under mechanical unloading conditions remains largely unexplored.

With growing interest in the gut-bone axis, the use of probiotics to mitigate osteoporosis has become a research hotspot. Several studies have demonstrated that supplementation with specific probiotic strains can significantly reduce trabecular bone loss and enhance BMD in the femur and vertebrae (22–24). Among these, *Lactobacillus rhamnosus* GG (LGG) has garnered considerable attention. Notably, LGG has been shown to modulate the human immune system and restore bone remodeling homeostasis in Ovariectomized (OVX) mice (12, 25, 26). Additionally, in mouse models of experimental periodontitis and drug-induced bone loss, LGG has also been demonstrated to suppress inflammation and attenuate bone loss (27, 28). However, the protective effect of LGG against bone loss induced by mechanical unloading has not been reported.

In this study, we hypothesized that LGG may attenuate bone loss by influencing gut microbiota composition, which could be associated with changes in SCFA production. Alterations in SCFA levels may contribute to improved intestinal barrier integrity and could be associated with changes in Treg cell abundance in the bone microenvironment, thereby reducing inflammatory stimuli and ultimately mitigating bone loss under mechanical unloading conditions. The present study aims to extend OVX-related findings to the microgravity context, elucidate the role of the gut-bone axis in unloading-induced bone loss, and propose novel strategies for bone health management in astronauts and bedridden populations.

## Materials and methods

### Animals

SPF grade male C57BL/6 mice (8 weeks old, 26 ± 2 g) were obtained from the Xi’an branch of Chongqing Tengxin Biotechnology Co., Ltd. (Xian, China). The experimental procedures were reviewed and authorized by the Ethics Committee of the 940th Hospital of the Joint Logistic Support Force (Approval No.: 2022KYLL021). All animals were kept under SPF conditions at 25 °C with a 12-h light/dark photo period. Following a 7-day adaptation period, animals were allocated to Control (Con), Hindlimb unloading (HU), and Hindlimb unloading with LGG treatment (HU + LGG) group. All analyses were conducted on samples collected from five randomly selected mice in each group.

### HU model

The HU model was developed following the protocol outlined by Qi et al. (29). Medical adhesive tape was wrapped around two-thirds of the tail length of each mouse, and a rotating tape was applied and fixed to maintain a head-down tilt position with hindlimbs suspended without weight-bearing, while forelimbs retained free movement. A schematic overview of the experimental workflow was provided in Supplementary Figure S1. After 1 week of environmental adaptation, mice in the HU and HU + LGG groups were subjected to HU for one additional week as a model acclimation phase. Both groups underwent continuous HU for 4 weeks. Simultaneously, the HU + LGG group received oral gavage of LGG suspension (1 × 10^9^ CFU per mouse), five times per week, for the entire 4-week period. The dose was selected based on previously published studies in which this concentration was shown to effectively modulate the gut microbiota and host physiology in mice without causing adverse effects (13). Due to experimental limitations, only a single dose was evaluated in the present study. Mice in the HU group were administered an equivalent volume of tap water, while those in the Con group remained untreated with ad libitum access to drinking water. After 4 weeks of intervention, all animals were humanely euthanized by rapid cervical dislocation performed by experienced personnel in strict accordance with the American Veterinary Medical Association (AVMA) Guidelines for the Euthanasia of Animals (2020). Death was confirmed by the absence of respiration, heartbeat, and pupillary reflex, after which samples were promptly collected for further analysis.

### 16S rRNA amplification and sequencing

Mouse fecal samples were collected using sterile disposable forceps and immediately transferred to −80 °C for storage. Microbial genomic DNA was isolated through the FastPure Stool DNA Isolation Kit (Majorbio, Shanghai, China). DNA integrity was verified by electrophoresis on a 1% agarose gel. The resulting DNA served as a template for amplifying the gene using primers 338F (ACTCCTACGGAGGCAGCAG) and 806R (GGACTACHVGGGTWTCTAAT). PCR products were examined by electrophoresis on a 1.5% agarose gel. Subsequently, the PCR products were purified using a QIAquick Gel Extraction Kit (Qiagen, Hilden, Germany). Then, the concentration of the purified PCR products was measured using a NanoDrop (Thermo Fisher Scientific, Waltham, MA, United States). Sequencing was conducted on the Illumina NextSeq 2000PE300 platform (Shanghai Meiji Biological Pharmaceutical Technology Co., Ltd., Shanghai, China). Raw fastq data will be made available upon request to the corresponding author.

### SCFA detection via LC-MS/MS

The SCFAs standard solutions were prepared. Under sterile conditions, small intestinal samples and serum were obtained. A 40 mg aliquot of intestinal tissue or 150 μL of serum was homogenized and mixed with an extraction solution (methanol:water = 4:1). The samples were ground using a cryogenic grinder, followed by ultrasonication under low temperature. The mixture was centrifuged at 13,000 rcf for 15 min at 4 °C. The supernatant was transferred into a new tube, followed by the addition of 20 μL of 3N HCl and 20 μL of EDTA HCl solution. The samples were incubated at 40 °C for 30 min, then diluted with an acetonitrile-water mixture to a final volume of 1,000 μL for analysis. After analysis, raw data were imported into the AB Sciex quantitative software system (AB Sciex, Framingham, MA, United States) for generating linear regression standard curves and calculating the sample concentrations.

### ELISA

Expression level of OCN, OPG, BALP, CTX, PINP, TNF-*α*, IL-1β, IL-10, and LPS were assessed via ELISA (Thermo Fisher Scientific, Waltham, MA, United States). Each group included five replicates, and each sample was measured in at least three technical replicates.

### Western blot (WB)

Total protein was isolated from colonic tissues and bone marrow, followed by SDS-PAGE. Membrane transfer was performed using a semi-dry method. Protein expression levels of ZO-1 and Occludin in colon tissues and TNF-α, IL-1β, and IL-10 in bone marrow were detected by WB. GAPDH was used as the internal reference. Primary antibodies, GAPDH (ab9485), ZO-1 (ab276131), Occludin (ab216327), TNF-α (ab183218), IL-1β (ab283818), and IL-10 (ab133575) were purchased from Abcam (Cambridge, United Kingdom). The secondary antibody used was goat anti-rabbit IgG (ab97051, Abcam, Cambridge, United Kingdom).

### Quantification of LGG by qPCR

Bacterial pellets from fecal samples were obtained according to previously described methods (30). Genomic DNA (gDNA) of LGG was isolated using the NucleoSpin kit (Takara, Kusatsu, Shiga, Japan). Quantitative polymerase chain reaction (qPCR) was performed to determine the abundance of LGG. Ct values of the samples were compared with a standard curve generated from pure cultures of LGG or fecal samples spiked with known concentrations of LGG, as previously described (30). The primers were listed in Supplementary Table S1.

### qRT-PCR

Primers for the Foxp3 and TLR4 gene in *Mus musculus* were designed based on its CDS sequence. The relative mRNA levels was carried out by CFX96 Touch Real-Time PCR Detection System (Bio-Rad Laboratories, Hercules, CA, United States). GAPDH was served as the internal control. Gene relative expression was assessed via the 2^−ΔΔCt^ approach. Each sample was assayed in at least three replicates. The primers were listed in Supplementary Table S2.

### Immunohistological analysis

The pre-section steps are similar to H&E staining, so only the post-section steps are covered. Deparaffinize and dehydrate the colon sections, perform antigen retrieval, block endogenous peroxidase activity, and incubate with rabbit serum at ambient temperature for 30 min. After rinsing, the sections were incubated overnight at 4 °C with ZO-1 (1:500) and Occludin (1:500) antibodies. Apply the secondary antibody (HRP-labeled), corresponding to the species of the primary antibody, and incubate at room temperature for 50 min. HRP-conjugated secondary antibodies corresponding to the host species of the primaries were applied and incubated at room temperature. Positive expression of ZO-1 and Occludin is considered positive when brown-yellow staining is detected. Finally, use ImageJ software to analyze the average optical density of all immunohistochemically stained sections.

### Femoral Tregs detection

Dissect the left femur of the mouse, remove any attached soft tissue, and sever both ends of the femur using bone scissors. Using a syringe, aspirate an appropriate volume of PBS and repeatedly flush the bone marrow cavity, collecting the released bone marrow cells into a centrifuge tube. Filter the solution through a 70 μm cell strainer, centrifuge the filtrate, and add red blood cell lysis buffer. Cells were subjected to three rounds of lysis to obtain a single-cell suspension. Take a small aliquot, stain with trypan blue, and enumerate the cells. Add 100 μL of the cell suspension into a centrifuge tube, followed by 5 μL each of CD45, CD3, CD4, and CD25 fluorescently labeled antibodies (BD Biosciences, San Jose, CA, United States) at the dose recommended by the manufacturer. Ensure thorough mixing, then incubate the samples in darkness at 4 °C for 30 min. Subsequently, centrifuge and remove the supernatant, followed by the addition of a cell membrane permeabilization reagent. Resuspend the cells gently, centrifuge once more, and discard the resulting supernatant. Repeat the resuspension using a permeabilization buffer for the fixed cells, centrifuge again, discard the liquid phase, and finally add 5 μL of Foxp3-specific antibody. Mix thoroughly and maintain in the dark at 4 °C for 50 min. After two resuspensions, analyze the cells using a flow cytometer and generate plots with CytExpert flow analysis software version CytExpert_Setup-2.5.0.77.

### Micro-CT scanning

The right femur of the mouse was dissected, the attached soft tissues were removed, and the specimen was fixed in 4% paraformaldehyde. Each experimental group consisted of five animals. Micro-CT scanning was performed as previously described (31, 32). Three-dimensional images of trabecular bone and corresponding microstructural parameters were subsequently obtained. The bone was removed from the 4% paraformaldehyde, gently dried with absorbent paper, wrapped in gauze, and subsequently placed into the scanning chamber. After scanning with Cruiser software, slice images were generated. The largest cross-sectional plane was designated for quantitative evaluation of trabecular parameters. The Avatar system was employed to analyze the morphological variations of trabecular architecture, focusing on the region 1–1.5 mm distal to the growth plate. Three-dimensional reconstructions of trabecular parameters, including trabecular bone density (Tb. BMD), trabecular surface area to tissue volume ratio (Tb. BS/TV), trabecular thickness (Tb. Th), trabecular volume fraction (Tb. BV/TV%), trabecular pattern factor (Tb. PF), trabecular separation (Tb. Sp), trabecular number (Tb. N), and trabecular surface area to volume ratio (Tb. BS/BV), were measured.

In addition, cortical bone parameters were evaluated in the femoral mid-diaphysis. Cortical thickness (Ct. Th), cortical area (Ct. Ar), and cortical bone mineral density (Ct. BMD) were quantified to assess cortical bone alterations.

### H&E staining

Dissect the right femur of the mouse, carefully remove the attached soft tissue, fix the sample in 4% paraformaldehyde, followed by dehydration, paraffin embedding, and sectioning. Stain the sections with H&E, and subsequently observe the area of the trabeculae. The trabecular bone area was analyzed using ImageJ. Within the same defined region, three fields of view were randomly selected for quantitative analysis.

### Statistical analysis

For microbial relative abundance data, log_10_ transformation was applied prior to statistical analysis to reduce skewness and approximate normal distribution. Chao1, Ace, Shannon, and Sobs were analyzed via the Kruskal–Wallis test. Beta diversity differences were assessed via principal coordinate analysis (PCoA) based on Bray–Curtis distance matrices and tested for significance using permutational multivariate analysis of variance (PERMANOVA).

In addition, other data were analyzed using SPSS 26.0. For group comparisons, the Shapiro–Wilk test was used to assess data normality, while Levene’s test assessed variance homogeneity. For data meeting parametric assumptions, one-way ANOVA followed by Tukey’s multiple comparisons was conducted. Non-parametric Kruskal–Wallis tests coupled with Dunn’s *post hoc* corrections were used for datasets violating parametric assumptions. In all statistical results, “*” indicates *p* < 0.05, “**” indicates *p* < 0.01, and “***” indicates *p* < 0.001.

## Results

### Supplementation with LGG remodels gut microbiota composition

**Figure 1:**
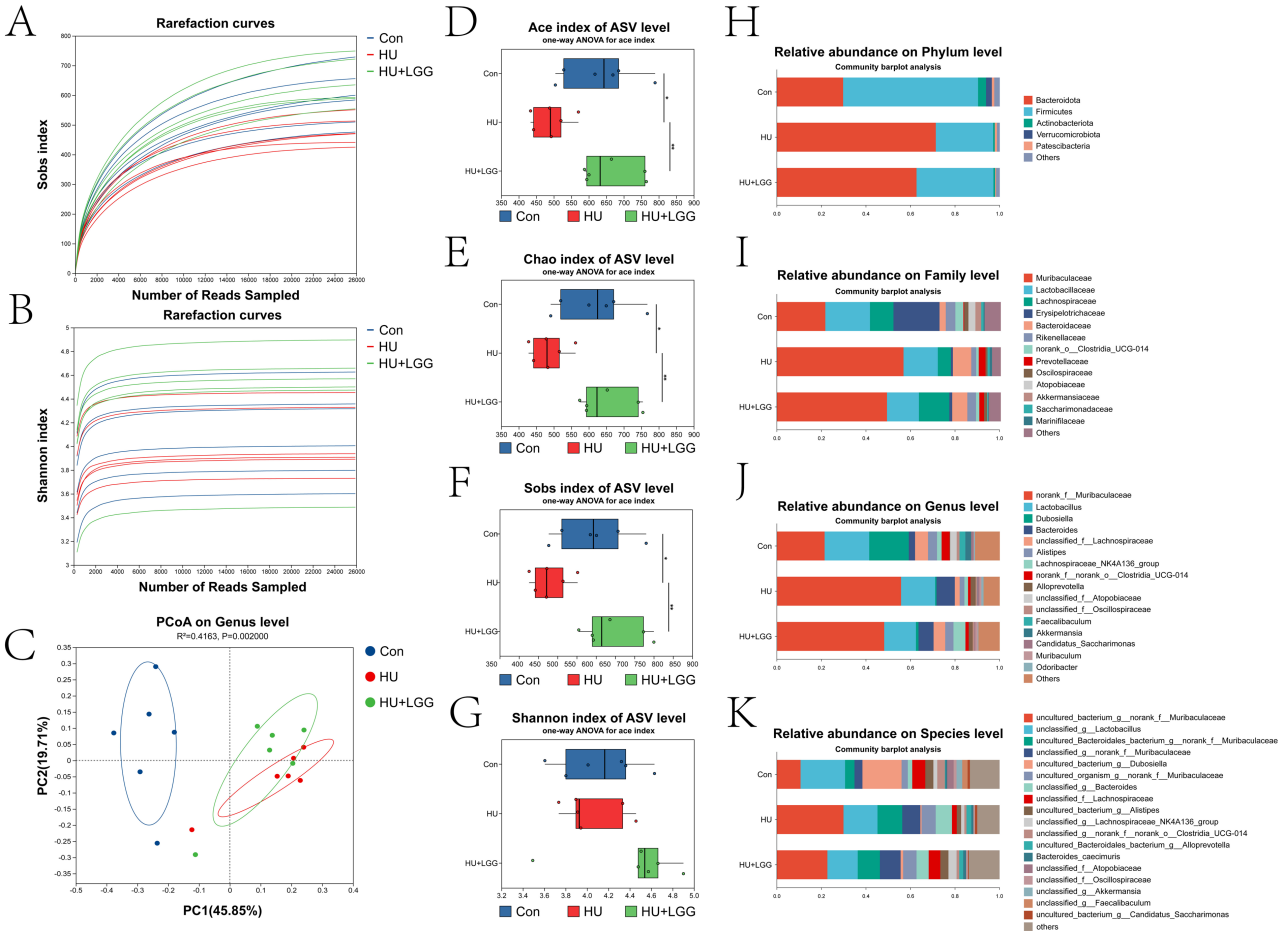
Analysis of gut microbial community structure via 16S rRNA sequencing. **(A,B)** Rarefaction plots generated using the Sobs and Shannon indices demonstrated that sequencing coverage was adequate to reflect the microbial diversity present in the samples. **(C)** Principal coordinate analysis (PCoA) was utilized to depict group-level differences in microbial community composition. **(D–G)** The Ace, Chao1, Sobs, and Shannon metrics applied to assess the richness and variety of the intestinal microbiota. **(H–K)** The taxonomic distribution of gut microbes at the phylum, family, genus, and species levels, respectively. Data are shown as mean ± SD. Each dot represents one sample. Statistical significance: *p < 0.05, **p < 0.01.

Gut microbiota dysbiosis has been shown to contribute to bone loss via the “gut-bone axis,” however, studies focusing on microbiota alterations under conditions of mechanical unloading remain limited. To elucidate changes in gut microbial structure under mechanical unloading and to determine whether LGG plays a role, 16S rRNA gene sequencing was conducted on fecal from mice in the Con, HU, and HU + LGG groups. Rarefaction curves indicated sufficient sequencing depth (Figures 1A,B). Principal coordinate analysis (PCoA) showed clear separation between Con and HU groups, while HU + LGG clustered closer to Con, suggesting partial restoration of microbial structure by LGG (Figure 1C and Supplementary Figure S2). HU significantly reduced *α*-diversity indices (Ace, Chao, and Sobs), indicating decreased species richness, whereas LGG supplementation reversed this decline (Figures 1D–G).

To more intuitively assess alterations in gut microbial composition, relative abundance was examined across phylum, family, genus, and species levels. At the phylum level, the proportion of *Bacteroidota* increased while *Firmicutes* and other phyla decreased in the HU group, most of which were partially reversed in the HU + LGG group (Figure 1H). Further analysis at the family and genus levels allowed for a more detailed understanding of microbial regulation by HU and LGG (Figures 1I,J). At the genus level, HU elevated *Muribaculaceae* and *Bacteroides* while reducing *Dubosiella* and *Lachnospiraceae*. These alterations were partially reversed following LGG treatment. At the species level, HU markedly elevated uncultured bacterium and Bacteroidales bacterium from the *Muribaculaceae* family, which were effectively reduced by LGG supplementation (Figure 1K).

### LGG promotes the metabolism of SCFAs

**Figure 2:**
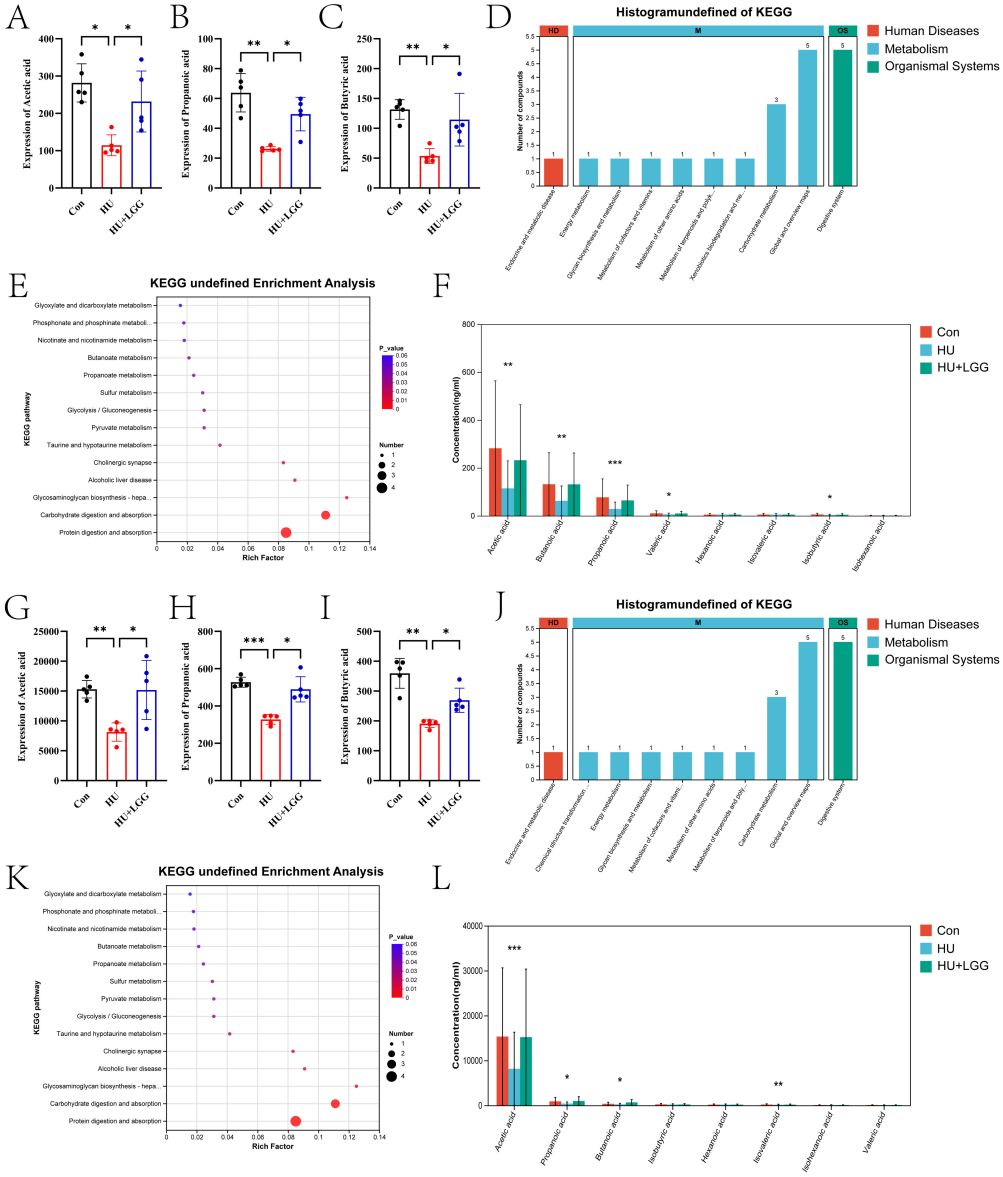
SCFA analysis in intestinal tissues and serum by LC–MS/MS. **(A–C)** Concentrations (ng/mL) of acetic, propanoic, and butanoic acid in serum. **(D,E)** KEGG pathway enrichment analyses of SCFAs in serum. **(F)** Composition of SCFAs in serum. **(G–I)** Concentrations (ng/mL) of acetic, propanoic, and butanoic acid in the small intestine. **(J,K)** KEGG pathway enrichment analyses based on small intestinal SCFA profiles. **(L)** Composition of SCFAs in the small intestine. Data are shown as mean ± SEM. Each dot in the statistical graph represents one sample. Statistical significance: *p < 0.05, **p < 0.01, ***p < 0.001.

To determine whether LGG modulates SCFA metabolism under mechanical unloading, LC–MS/MS was used to quantify SCFAs in serum and intestinal tissues. Mechanical unloading significantly reduced acetic, propanoic, and butanoic acid levels, whereas LGG supplementation restored these metabolites in both serum and intestine (Figures 2A–C,G). KEGG pathway enrichment revealed that SCFAs were mainly involved in metabolic processes, including carbohydrate and energy metabolism, and were enriched in the “protein digestion and absorption” pathway, indicating their importance in nutrient processing (Figures 2D,E,J,K). Acetic, propanoic, and butanoic acids were the predominant SCFAs, with no significant differences in minor acids such as hexanoic or isovaleric acid among groups (Figures 2F,L).

### LGG restores intestinal barrier integrity under mechanical unloading

**Figure 3:**
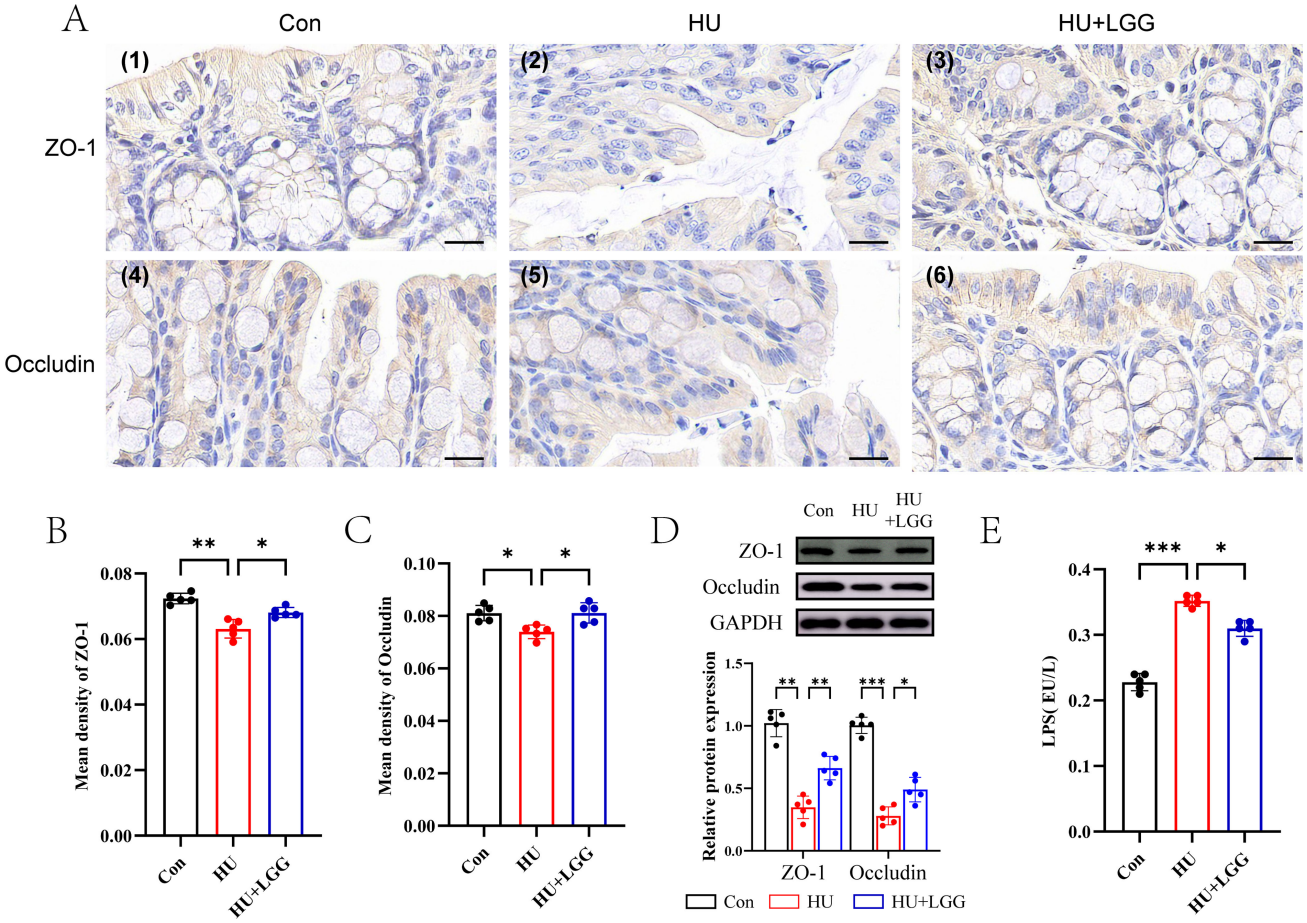
Assessment of intestinal barrier integrity and function. **(A)** Immunohistochemical detection of ZO-1 and occludin proteins in colon sections. Black scale bar represents 10 μm. **(B,C)** Densitometric quantification of ZO-1 and occludin staining intensity based on immunohistochemical images. **(D)** Western blot analysis of ZO-1 and occludin protein expression. **(E)** Enzyme-linked immunosorbent assay (ELISA) used to quantify circulating LPS concentrations in serum. Data are shown as mean ± SEM. Each dot represents one sample. Statistical significance: *p < 0.05, **p < 0.01, ***p < 0.001.

LGG abundance in fecal samples was first quantified by qPCR. The results showed that LGG was detectable in the HU + LGG group, with levels ranging from approximately 10^4^ to 10^6^ CFU per gram of feces, whereas LGG was not detected in fecal samples from the Control and HU groups (Supplementary Figure S3). Given the critical role of the intestinal barrier in regulating systemic inflammation and nutrient absorption, immunohistochemistry and WB were performed to detect the expression of ZO-1 and Occludin in colonic tissues. The results demonstrated that HU significantly reduced the protein expression of ZO-1 and Occludin, with mean optical density values significantly decreased (*p* < 0.05), indicating compromised intestinal barrier integrity and increased intestinal permeability (Figure 3A). Following oral LGG supplementation, the levels of ZO-1 and Occludin were markedly restored, indicating enhanced intestinal barrier integrity and decreased epithelial permeability (*p* < 0.05) (Figures 3B–D).

LPS, a marker of bacterial endotoxin, is known to provoke inflammatory responses in the bone microenvironment, contributing to bone loss (33, 34). Increased intestinal permeability may facilitate LPS translocation into the serum, potentially aggravating inflammatory infiltration and osteolysis. HU markedly increased serum LPS levels (*p* < 0.001), consistent with compromised barrier function and elevated permeability (Figure 3E). LGG supplementation significantly reduced serum LPS concentrations, further supporting its protective role in barrier integrity. Consistently, qRT-PCR analysis showed that the mRNA expression level of TLR4 was significantly increased in the HU group (*p* < 0.001), whereas LGG treatment partially reversed this increase (*p* < 0.05, Supplementary Figure S4).

### LGG promotes bone marrow Treg cell expansion and suppresses inflammatory responses

**Figure 4:**
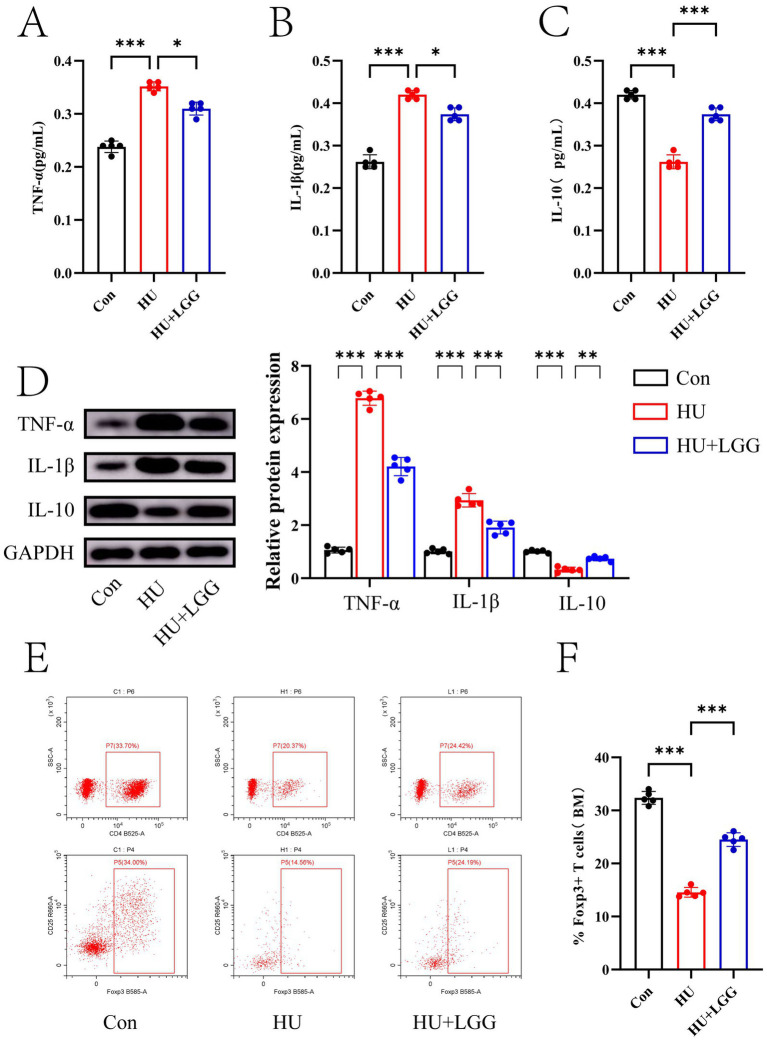
Expression of inflammatory markers and proportions of Treg cells. **(A–C)** ELISA quantification of TNF-α, IL-1β, and IL-10 in the bone microenvironment. **(D)** WB analysis of inflammatory cytokine expression. **(E)** Flow cytometric detection of Treg cell proportions in Con, HU, and HU + LGG groups. **(F)** Statistical analysis of flow cytometry results. Data are presented as mean ± SEM. Each dot represents one sample. Statistical significance: *p < 0.05, **p < 0.01, ***p < 0.001.

To explore whether LGG-induced improvements in gut and metabolic function could modulate inflammation in the bone microenvironment, the levels of key inflammatory markers and the proportion of Treg cells in bone marrow were assessed. ELISA results revealed that HU significantly elevated TNF-α and IL-1β, while reducing IL-10 (*p* < 0.001) (Figures 4A–C). This was corroborated by WB, which confirmed increased TNF-α and IL-1β protein levels and decreased IL-10 expression following HU (Figure 4D). These findings, in line with elevated serum LPS levels and impaired intestinal barrier function, indicate that HU provokes inflammatory responses in the bone microenvironment. LGG intervention significantly suppressed TNF-α and IL-1β (*p* < 0.05) expression while restoring IL-10 levels (*p* < 0.001), thereby rebalancing the inflammatory milieu.

Considering the critical function of Treg cells in modulating immune suppression and influencing bone remodeling, flow cytometry was employed to quantify CD4^+^CD25^+^Foxp3^+^ Treg cells in bone marrow (Figures 4E,F and Supplementary Figure S5) (35, 36).

### LGG improves skeletal microarchitecture and bone metabolism

**Figure 5:**
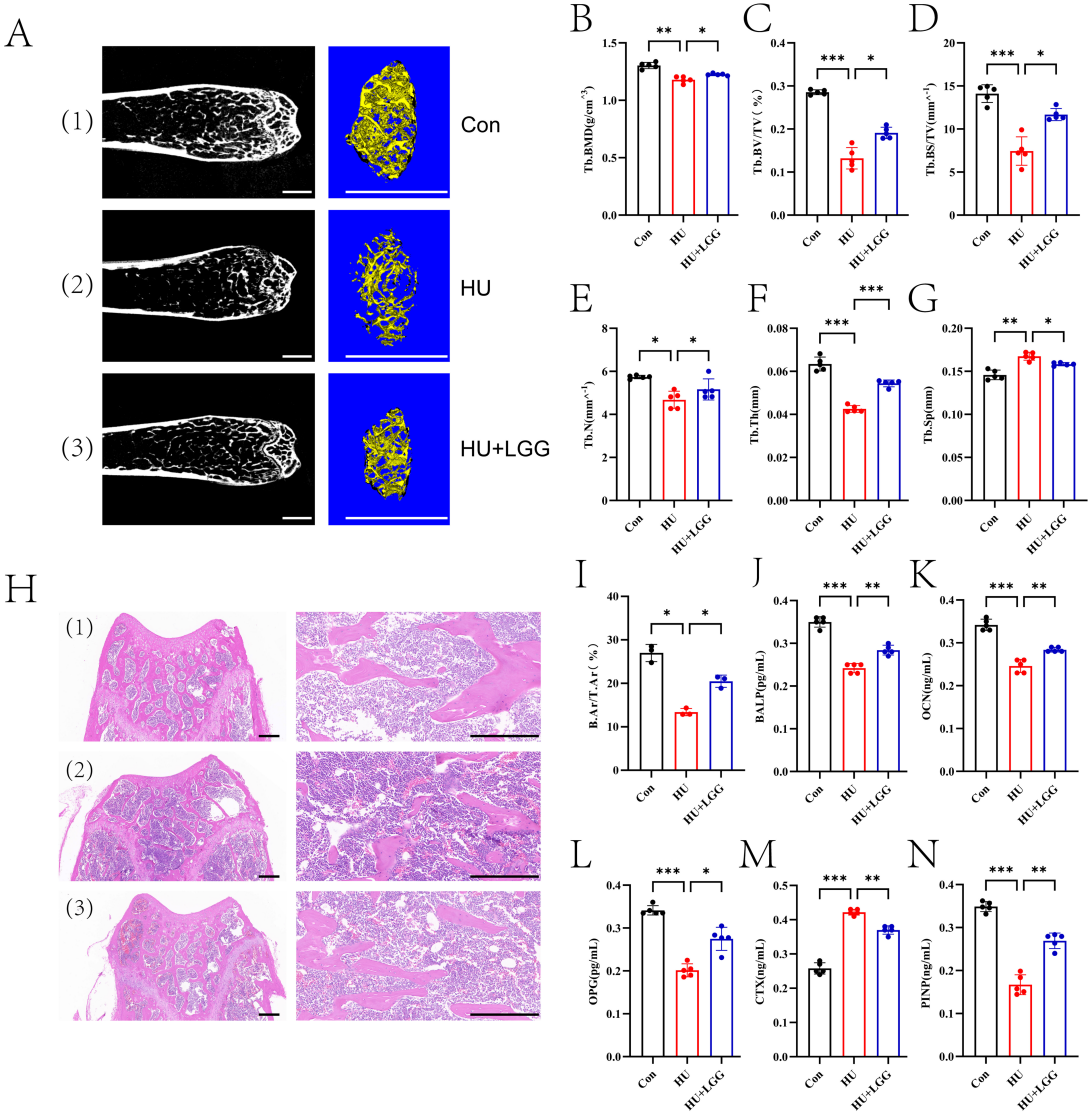
Detection of trabecular damage and related indicators. **(A)** Micro-CT visualization of trabeculae. White scale bar: 1 mm. **(B–G)** Quantitative analysis of BMD, BV/TV, BS/TV, Tb. N, Tb. Th, and Tb. Sp in trabecular bone. **(H)** H&E staining of trabecular bone. Black scale bar: 200 μm. **(I–N)** Quantification of B. Ar/T. Ar, BALP, OCN, OPG, CTX, and PINP. Data are presented as mean ± SEM. Each dot represents one sample. Statistical significance: *p < 0.05, **p < 0.01, ***p < 0.001.

To determine whether LGG could alleviate bone deterioration induced by mechanical unloading, key trabecular parameters of the distal femur were analyzed. Histological evaluation was performed, and bone metabolic markers in the bone marrow were quantified. Micro-CT results (Figures 5A–G) indicated that trabecular bone in the HU group was substantially reduced, as reflected by significantly decreased values of BMD, BV/TV, BS/TV, Tb. N, and Tb. Th, along with a notable rise in Tb. Sp (*p* < 0.05). Oral administration of LGG was found to improve these parameters, indicating that LGG supplementation could mitigate HU-induced trabecular damage. These findings were corroborated histologically by H&E staining, which revealed a pronounced reduction in trabecular area and sparse bone matrix in the HU group (Figures 5H,I), whereas LGG-supplemented mice exhibited denser trabecular structures and more regular architecture. HU also induced significant changes in cortical bone microarchitecture, which were partially attenuated by LGG treatment (Supplementary Figure S6).

To gain deeper insights into the influence of LGG on bone remodeling balance, serum levels of BALP, OPG, OCN, CTX, and PINP were measured (Figures 5J–N). HU significantly decreased BALP, OPG, OCN, and PINP levels while markedly increasing CTX levels, indicating reduced osteoblast activity and enhanced osteoclast-mediated bone resorption, thereby disrupting bone remodeling homeostasis. Notably, LGG supplementation was able to modulate the differentiation and maturation of both osteoblasts and osteoclasts, as evidenced by the restorative elevation of BALP, OPG, OCN, and PINP levels, and a concurrent reduction in CTX concentration.

## Discussion

Bone loss induced by mechanical unloading poses a significant health risk to long-term bedridden patients and astronauts exposed to microgravity environments. Although probiotic strategies targeting the gut microbiota have shown promise in regulating bone metabolism, their role under unloading conditions remains unclear. This study employed a HU mouse model to explore the gut-bone axis in the context of mechanical unloading.

Intestinal microbiota is recognized as key regulators of host metabolic processes. Alterations in microbial composition, particularly the reduction of beneficial anti-inflammatory bacteria and the increase in opportunistic pathogens, have been shown to affect immune responses and impair the overall health of astronauts during spaceflight (37). The HU model revealed significant alterations in gut microbiota, notably an increase in *Bacteroidota* and a decrease in *Firmicutes*. The ratio of *Firmicutes* to *Bacteroidota* (F/B), widely regarded as a marker of gut metabolic balance, is closely associated with nutrient absorption and energy harvesting efficiency (38–41). A reduction in F/B ratio observed in the HU group indicates a disturbance in gut metabolic homeostasis, which may contribute to systemic consequences (42). Moreover, HU led to a significant decrease in beneficial bacteria such as *Dubosiella*, *Lachnospiraceae*, and *Clostridium*. *Dubosiella*, known for its protective effects against ulcerative colitis and its ability to enhance the intestinal barrier, has also been reported to alleviate osteoporosis by correcting metabolic disturbances. Notably, *Dubosiella*, *Lachnospiraceae*, and *Clostridium* are major producers of SCFAs (43–45). The reduction in their abundance may thus be a key factor underlying the reduction of SCFA levels observed under HU conditions (46). These findings demonstrate that mechanical unloading via hindlimb suspension profoundly disrupts gut microbial homeostasis and may affect intestinal or systemic SCFA levels.

Short-chain fatty acids, primarily acetic, propanoic, and butanoic acid, are key mediators of the gut-bone axis (47–49). SCFAs serve as critical signaling molecules linking the gut microbiota with inflammatory responses and bone metabolic homeostasis. In models of periodontitis, rheumatoid arthritis, and metabolism-associated bone loss, SCFAs have been shown to significantly suppress the expression of pro-inflammatory cytokines, ameliorate both local and systemic inflammatory states, thereby attenuating bone resorption and promoting the restoration of bone homeostasis (50, 51). Furthermore, SCFAs have been reported to influence the bone-immune microenvironment by regulating immune cell metabolism and differentiation (24). Under conditions of stress or metabolic dysregulation, reductions in SCFA levels are frequently accompanied by activation of inflammatory signaling pathways and exacerbation of bone loss (52). Collectively, these findings indicate that SCFAs act as a critical mechanistic bridge between inflammatory regulation and metabolic bone loss. In addition, SCFAs help regulate immune homeostasis by modulating G protein-coupled receptors (GPRs), thereby balancing pro- and anti-inflammatory responses (53–55). Specifically, SCFAs promote osteogenesis and modulate inflammation through the inhibition of histone deacetylases and the expansion of Treg cells (56, 57). In particular, butyrate supplementation has been reported to increase Treg abundance and induce Wnt10b expression in CD8^+^ T cells, thereby suppressing bone resorption (13, 58). Our findings revealed a marked reduction in Treg cell proportions under mechanical unloading, suggesting that SCFAs may influence inflammatory responses and bone homeostasis via Treg regulation under such conditions.

In addition, certain gut microbes, such as members of the *Muribaculaceae* family, can secrete LPS, leading to chronic intestinal inflammation and barrier dysfunction (59, 60). Our microbiota analysis revealed a significant increase in *Muribaculaceae* and *Bacteroides* in the HU group, which may exacerbate intestinal inflammation and compromise epithelial barrier integrity. Consistently, we observed altered expression of colonic ZO-1 and Occludin, suggesting increased intestinal permeability under HU conditions (61, 62). This may facilitate the translocation of pathogenic factors such as LPS into systemic circulation, thereby provoking inflammatory responses (63). In the HU group, significantly elevated levels of TNF-α and IL-1β, along with a marked reduction in IL-10, were observed—indicative of an inflammatory response. These observations suggest that alterations in Treg cell proportions and elevated serum LPS levels may contribute to a pro-inflammatory milieu. Inflammation is recognized as a major determinant of bone loss, as it fundamentally influences bone metabolism, leading to reduced bone mass and increased fracture risk (64). Consistent with this, HU mice exhibited decreased BMD and disrupted trabecular architecture. Moreover, bone formation markers (BALP, OCN, and PINP) and the bone resorption marker (CTX) were significantly altered, indicating that mechanical unloading ultimately caused an imbalance in bone homeostasis and accelerated bone loss (65).

The LGG is a widely studied probiotic. Early-life colonization with LGG has been reported to upregulate the population of SCFA-producing bacteria and reduce inflammation (66). In addition, LGG can regulate the expression of ZO-1 and Occludin, thereby alleviating barrier dysfunction (67), suggesting its essential role in protecting against inflammatory damage and preserving intestinal barrier integrity. In our study, oral administration of LGG in HU mice significantly reshaped the gut microbiota, partially restoring the F/B ratio and increasing the abundance of SCFA-producing genera. We also observed elevated SCFA levels in both serum and small intestine following LGG treatment, indicating that LGG may enhance SCFA synthesis and transport by modulating microbial composition. Furthermore, the increased SCFA levels contributed to the recovery of tight junction protein expression, potentially preventing LPS translocation into the bloodstream. SCFAs also promoted the restoration of Treg cell proportions. Collectively, these effects may ultimately lead to increased BMD and improved trabecular microarchitecture in LGG-treated mice.

### Limitations

The HU mouse model is a widely used animal model for simulating mechanical unloading, such as that induced by microgravity or prolonged bed rest. However, its translational applicability to human physiology remains limited. Additionally, the use of LGG in this study was restricted to animal models, and its clinical relevance has yet to be evaluated. While the biosafety of LGG has been partially demonstrated, further comprehensive validation of its safety profile may be of great importance for future clinical translation. Moreover, most experimental groups in the present study consisted of five biological replicates, which may reduce statistical power and should be considered when interpreting the results.

This study did not include a separate healthy + LGG (positive control) group, as our primary objective was to investigate the protective effects of LGG under mechanical unloading conditions, rather than its baseline influence under normal physiological states. Previous studies have consistently shown that LGG does not exert adverse effects on skeletal parameters in healthy animals. Nevertheless, the lack of a positive control group may still be considered a limitation, potentially restricting the interpretation of LGG’s independent effects unrelated to unloading. More rigorous experimental grouping will be considered in future mechanistic investigations.

This study primarily focused on phenotypic observations of bone loss modulation via the gut-bone axis under HU and LGG intervention. Nevertheless, the core molecular pathways governing this regulatory process have yet to be elucidated. Additional studies are warranted to delineate the specific signaling pathways involved in this axis.

## Conclusion

In conclusion, our study demonstrates that LGG effectively alleviates bone loss induced by mechanical unloading, an effect that may be attributed to its ability to remodel the gut microbiota, thereby restoring SCFA levels and repairing intestinal barrier integrity. These improvements further enhance Treg cell activation and reduce systemic inflammation. These findings highlight the potential involvement of the gut-bone axis in unloading-induced osteoporosis and support the therapeutic potential of LGG as a microbiota-targeted intervention to maintain bone health under conditions of microgravity or prolonged immobility. Nevertheless, additional mechanistic and translational studies are warranted to validate the clinical applicability of LGG and to explore its incorporation into countermeasure strategies for astronauts and bedridden populations.

## Data Availability

The original contributions presented in the study are included in the article/Supplementary material, further inquiries can be directed to the corresponding authors. The raw reads were deposited in the Sequence Read Archive (SRA) database (SRA accession: PRJNA1399705).
