# Supplementary material for: Lactobacillus rhamnosus GG mitigates bone loss induced by mechanical unloading via regulation of the gut-bone axis
Source: Front Nutr. 2026 Feb 2;12:1734220. doi: 10.3389/fnut.2025.1734220 (PMC12907305; doi:10.3389/fnut.2025.1734220)
Supplement: Supplementary file 1 [file Table_1.docx]

Supplementary Materials

Supporting Table

Table S1. Primers for LGG

| Name | Primers (5’-3’) |
| --- | --- |
| LGG-F | CGCCCTTAACAGCAGTCTTC |
| LGG-R | GCCCTCCGTATGCTTAAACC |

Table S2. Primers for qRT-PCR

| Name | Primers (5’-3’) |
| --- | --- |
| Foxp3-F | ACCCAGGAAAGACAGCAACC |
| Foxp3-R | CTCGAAGACCTTCTCACAACCA |
| TLR4-F | AGTGGGTCAAGGAACAGAAGCA |
| TLR4-R | CTTTACCAGCTCATTTCTCACCC |
| GAPDH-F | AGGTCGGTGTGAACGGATTTG |
| GAPDH-R | TGTAGACCATGTAGTTGAGGTCA |

Supporting Figures


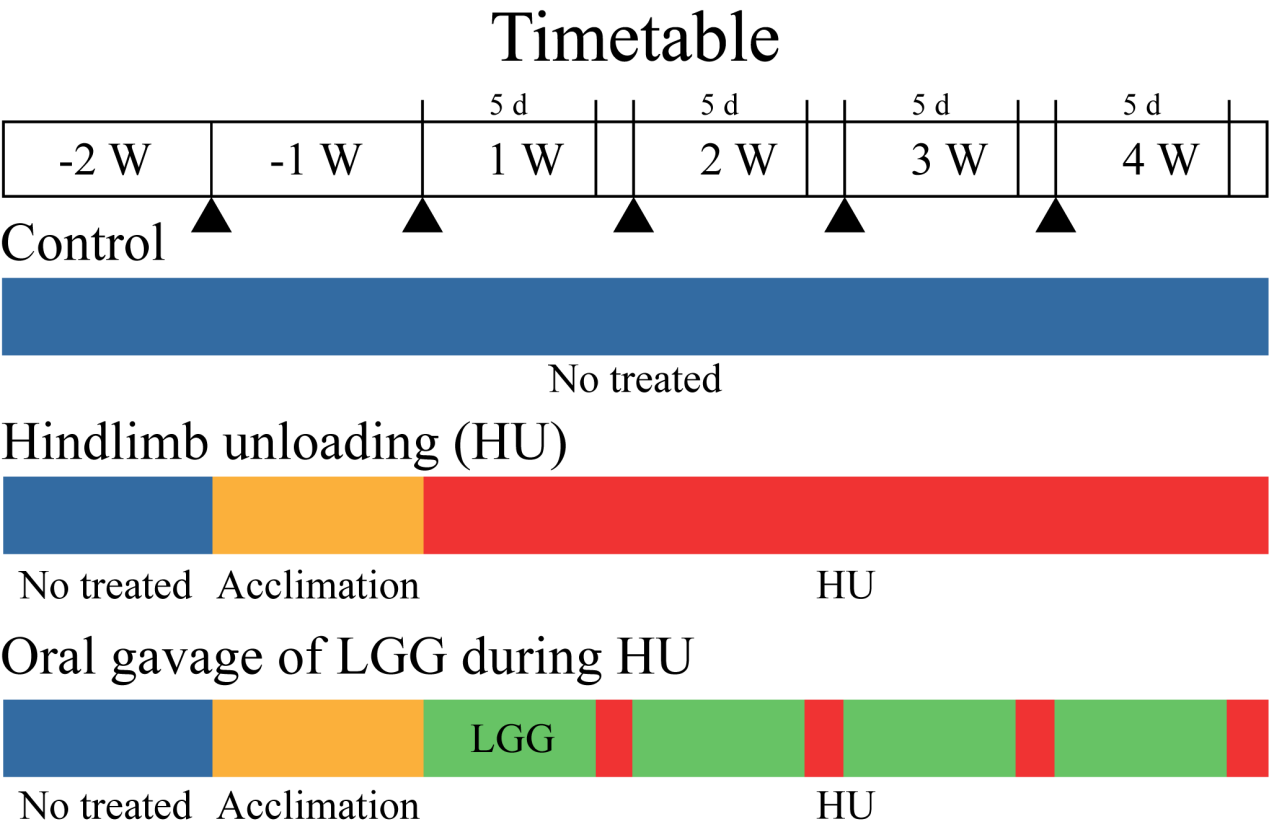


Figure S1. Experimental timeline and intervention schedule for the animal study.

Mice in the Control group received no intervention. Mice in the HU group underwent one week of acclimation to the housing environment, followed by one week of acclimation to the HU condition, and were then subjected to continuous hindlimb unloading for 4 weeks. Based on the HU protocol, mice in the HU+LGG group received oral gavage of LGG five times per week for 4 consecutive weeks.


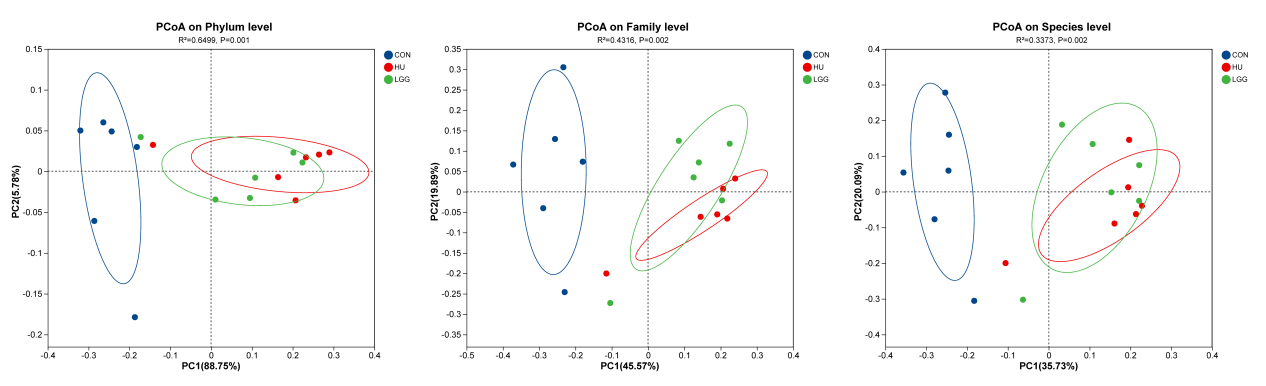


Figure S2. PCoA results based on 16S rRNA sequencing data.

PCoA was performed at the phylum, family, and species levels, respectively.


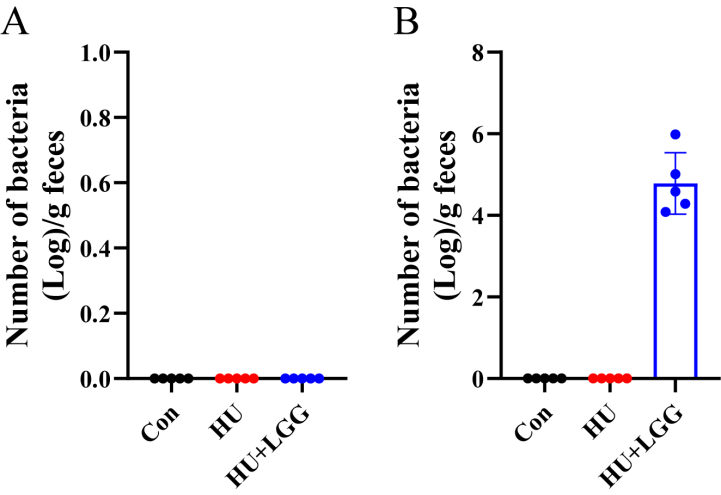


Figure S3. Quantification of LGG levels in fecal samples.

(A) Abundance of Lactobacillus rhamnosus GG (LGG) in fecal samples collected on day 0. (B) Abundance of LGG in fecal samples collected on day 28. Each dot represents one sample.


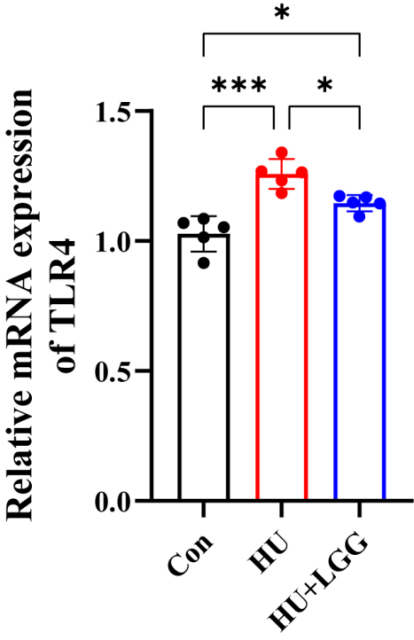


Figure S4. Statistical analysis of relative mRNA expression levels of TLR4.

The relative mRNA expression level of TLR4 in bone marrow was measured to assess the involvement of LPS-related signaling in local inflammatory responses.Data are shown as mean±SEM. Each dot represents one mouse. **P* < 0.05, ****P* < 0.001.


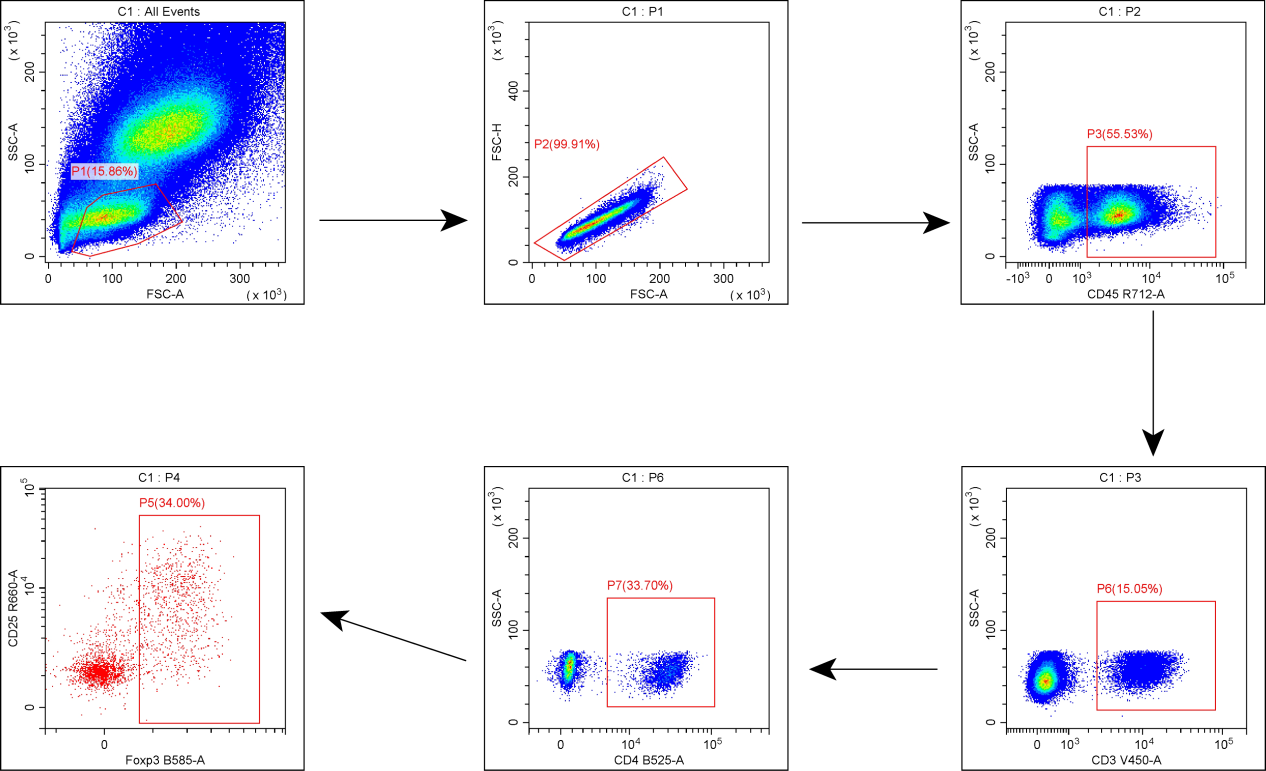


Figure S5. Gating strategy used for flow cytometric analysis.


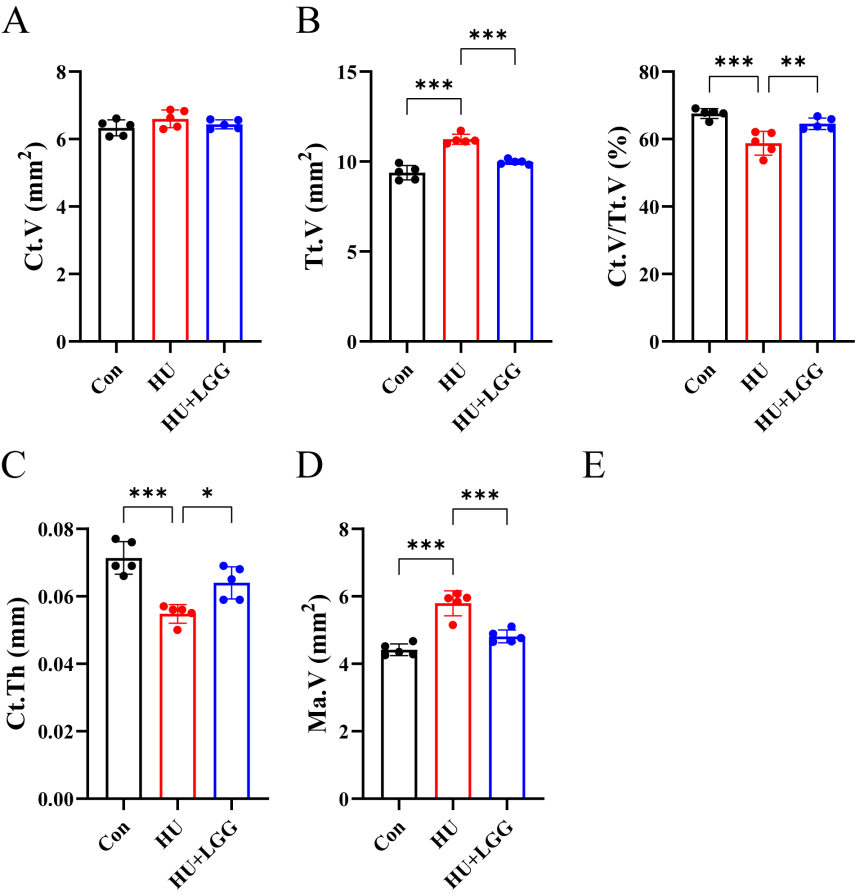


Figure S6. Quantitative analysis of cortical bone microarchitectural parameters assessed by micro-CT.

(A-E) Ct.V, Tt.V, Ct.V/Tt.V, Ct.Th, and Ma.V, respectively. Data are shown as mean±SEM. Each dot represents one mouse. **P* < 0.05, ***P* < 0.01, ****P* < 0.001.
